# Pesticide poisoning in nonfatal, deliberate self-harm: A public health issue

**DOI:** 10.4103/0019-5545.33259

**Published:** 2007

**Authors:** A. N. Chowdhary, Sohini Banerjee, Arabinda Brahma, M. K. Biswas

**Affiliations:** Institute of Psychiatry, 7 D. L. Khan Road, Kolkata - 700 025, India

**Keywords:** Community psychiatry, deliberate self-harm, pesticide poisoning, primary care, Sundarban

## Abstract

**Background:**

Nonfatal, deliberate self-harm (DSH), particularly with pesticides, is a major public health problem in many developing countries of the world. Agriculture is the primary occupation of most people living in the Sundarban region in West Bengal, India. Pesticides are extensively used in agriculture and these agents are most frequently used in DSH.

**Aim:**

This study sought to identify the nature of methods and agents used in nonfatal DSH attempts in the Sundarban area under South 24 Parganas district of West Bengal.

**Materials and Methods:**

Detailed demographic and clinical data on DSH cases of 13 Block Primary Health Centres (BPHCs') admission registers were analysed. Focus Group Discussions (FGDs) were conducted with the Panchayat Samithy of each block to elicit their perception about the problem of pesticide-related DSH or suicide in the region.

**Results:**

Five thousand, one hundred and seventy-eight (1,887 male and 3,291 female) subjects were admitted in the BPHCs during the study period from 1999 to 2001. Organophosphorous pesticide poisoning was found to be the most common method (85.1%) in DSH. This emphasizes the importance of developing an urgent poisoning prevention program with a special focus on improving clinical services as well as initiating farmers' education programs focusing on safe pesticide practices at the primary care level.

Deliberate self-harm (DSH), particularly with pesticides, is a major public health problem in the developing countries.[1–3] Some studies from India have reported similar findings,[4–7] although documentation on this issue is often not available from very remote areas such as the Sundarban region in West Bengal, India. Sundarban is the largest delta in the world and famous for its mangrove vegetation and Royal Bengal tigers. Indian Sundarban is the southern most part of the state of West Bengal at the confluence of the river Hooghly and the Bay of Bengal.

The Sundarban region in the South 24 Parganas district has 13 community development blocks (CDBs). Each CDB has an average population of 1-1.5 lakhs (1 lakh = 10^5^) and a nodal health service facility known as the Block Primary Health Centre (BPHC). Sundarban is a backward region by all yardsticks of socio-economic development. The literacy rate and per capita income is much lower than the state average and about 88.5% inhabitants are dependent on agriculture. Life in the entire region is subject to various ecological hazards. Coastal erosion, tidal ingression, sand encroachment and salinity of soil are the few factors that adversely affect agriculture.[8]

In the course of a previous mental health research study in the Sundarban region, the local community expressed concerns about DSH and suicide particularly with pesticides. To study the extent of DSH in the region, research was carried out in all the BPHCs of the 13 CDBs, *viz*, Basanti, Canning I and Canning II, Gosaba, Joynagar I, Joynagar II, Kakdwip, Kultali, Mathurapur I, Mathurapur II, Namkhana, Patharpratima and Sagar.

## MATERIALS AND METHODS

### DSH data:

The research team made 3-4 visits to each of the 13 BPHCs between January and April 2002. Information on DSH was collected for the period from 01.01.99-31.12.01 from admission and emergency registers. Data on suicides and accidental poisoning was also collected which have been reported elsewhere. This article discusses the different types of agents used in DSH.

### Participatory Research observation:

Thirteen Focus Group Discussions (FGDs) were conducted with the Panchayat Samithy of each block. Their opinions about socio-cultural dynamics of DSH and pesticide use were collected. The Panchayat Samithy is the locally elected democratic body, which acts as the local administrative organ in the three-tier Panchayat system, responsible for implementation of all developmental work for the blocks including health services.

## RESULTS

There were 1691 cases (male 579, female 1112) of DSH in 1999, 1775 cases (male 668, female 1107) of DSH in 2000 and 1712 cases (male 640, female 1072) of DSH in 2001. Table 1 shows the sex-wise distribution of all the combined cases (5178 cases: 1887 male, 3291 female) according to the BPHCs. The maximum number of cases was admitted in Joynagar I (1286) followed by Canning I (898) BPHC. Females (63.6%) inflicting DSH have a clear predominance over males (36.4%) in all the three years.

**Table 1:** Block primary health centres-wise distribution of methods in nonfatal deliberate self-harm cases (1999-2001)

Blocks/DSH number	Male							Female							
		
	Hang	Burn	Drown	HCh	IP	OP	Oth	Hang	Burn	Drown	Inj	HCh	IP	OP	Oth
Basanti	1	1				7		1	1				1	12		
n = 24 (M:9/F: 15)	11.1	11.1				77.8		6.7	6.7				6.7	80.0		
Canning I	7	12	1	5	6	272	18	3	25	1		10	18	502	18	
n = 898 (M:321/F:577)	2.2	3.7	0.3	1.6	1.9	84.7	5.6	0.5	4.3	0.2		1.7	3.1	87.0	3.1
Canning n		1				10			1			1	2	18		
n = 33 (M: 11/F:22)		9.1				90.9			4.5			4.5	9.1	81.8		
Gosaba		2				29			4			2	1	44		
n = 82 (M:31/F:51)		6.5				93.5			7.8			3.9	2.0	86.3		
Jaynagar I		2		4	1	446	4	1	2			10	4	803	9
n= 1286 (M:457/F: 829)		0.4		0.9	0.2	97.6	0.9	0.1	0.2			1.2	0.5	96.9	1.1
Jaynagar n	3			3		84	28	1	1	1		6	3	140	40
n = 310 (M: 118/F: 192)	2.5			2.5		71.2	23.7	0.5	0.5	0.5		3.1	1.6	72.9	20.8
Kakdweep	7	5	2	4	5	166	9	4	10	1		19	16	267	8
n = 523 (M:198/F:325)	3.5	2.5	1.0	2.0	2.5	83.8	4.5	1.2	3.1	0.3		5.8	4.9	82.2	2.5
Kultali						32	7						1	89	24
n=153 (M:39/F: 114)						82.1	17.9						0.9	78.1	21.1
Mathurapur I	2	5	1	7	2	146	5	2	4			15	7	245	27
n = 468 (M: 168/F: 300)	1.2	3.0	0.6	4.2	1.2	86.9	3.0	0.7	1.3			5.0	2.3	81.7	9.0
Mathurapur n	2	2	1	4		193	6	2	9	2	1	8	3	291	11
n = 535 (M:208/F:327)	1.0	1.0	0.5	1.9		92.8	2.9	0.6	2.8	0.6	0.3	2.4	0.9	89.0	3.4
Namkhana	1	1			2	80	24	3	1			3	30	114	23
n = 282 (M: 108/F: 174)	0.9	0.9			1.9	74.1	22.2	1.7	0.6			1.7	17.2	65.5	13.2
Patharpratima		1		4	1	71	22	1	1	1		5	8	114	36
n = 265 (M:99/F: 166)		1.0		4.0	1.0	71.7	22.2	0.6	0.6	0.6		3.0	4.8	68.7	21.7
Sagar				12	5	97	6	1				32	22	135	9
n = 319 (M:120/F:199)				10.0	4.2	80.8	5.0	0.5				16.1	11.1	67.8	4.5
Total	23	32	5	43	22	1633	129	19	59	6	1	111	116	2774	205
n = 5178 (M:1887/F: 3291)	1.2	1.7	0.3	2.3	1.2	86.5	6.8	0.6	1.8	0.2	0.03	3.4	3.5	84.3	6.2

Hch = Household chemicals, IP = Indigenous Poisons, OP = Organophosphorus, Inj = Injury (Self-inflicted), Oth = Others

Figures in the second row in each box represent percentages.

Table 2 shows the nature of different methods / agents used in DSH attempts. Poisoning, especially by organophosphorous pesticides, was the most common (85.1%) with a slight male preponderance (86.5%) over females (84.3%). The miscellaneous category termed ‘Others’ (Unknown Poisoning and Medicines like Sedatives, Homeopathic medicines etc.) comes next (6.5%) followed by household chemicals (3.0%) like kerosene, rat killer, lice killer etc. and indigenous poisons (2.7%) like oleander seeds, Datura seeds etc. Traditional methods like self-immolation (1.8%), hanging (0.8%) and drowning (0.2%) were attempted by very few subjects.

**Table 2:** Distribution of methods / agents of nonfatal deliberate self-harm cases (1999-2001)

Method/agents	Male		Female		Total	
			
	n = 1887	%	n = 3291	%	n = 5178	%
Pesticide poisoning	1633	86.5	2774	84.3	4407	85.1
Burning	32	1.7	59	1.8	91	1.8
Drowning	5	0.3	6	0.2	11	0.2
Household chemical	43	2.3	111	3.4	154	3.0
Hanging	23	1.2	19	0.6	42	0.8
Indigenous poisoning	22	1.2	116	3.5	138	2.7
Self injury		0.0	1	.03	1	.02
*Other	129	6.8	205	6.2	334	6.5

* Prescribed medicine, alcohol, unknown poisoning

Table 3 shows the summary findings from the FGDs from 13 Panchayat Samities, which mostly highlight the issue of DSH with pesticide poisoning as an emerging public health problem in the region. They also endorsed the opinion that varied types of psychosocial stressors, especially among young married females are positively linked with suicidal behavior. All Panchayat Samities felt the need for greater empowerment of the Panchayat to supervise the local trade of pesticides along with imposition of stricter regulations on pesticide sale by the administration.

**Table 3:** Summary findings of the FGDs with Panchayat Samithies

Deliberate self-harm and suicide by pesticide poisoning is quite common in the block and it has become a major health problem in the entire Sundarban region. Although it is seen in both sexes, the frequency of DSH is higher among females, specially among young married females. Torture is an important cause behind many deliberate self-harm attempts in women. Family conflict and economic loss may be important reasons among males. Recently, deliberate self-harm has been observed among schoolgoing boys and girls, mostly related to failure in (love) relationships or examinations. Exposure of youth to sex and violence in movies, in video parlors is highly influential in the development of the habit to consume alcohol, indecent sexual behavior and torture and violence against women. Easy availability of pesticides is a dangerous situation prevailing in this entire region. Farmers' education on safe use and storage of pesticides is an important method of public awareness. Panchayats should have some power to regulate the local pesticide market. There is a definite lack of appropriate caution or knowledge of the safe storage of pesticides by farmers. Opportunities for health care service should be available at the community level. Timely family intervention may save many lives if arranged. Governmental intervention concerning pesticide licensing or legal proceedings in cases of dowry-related deliberate self-harm and suicide should be handled more strictly.

## DISCUSSION

In many Asian countries, hanging, self-immolation and jumping from heights are common methods of self-harm acts.[9] However, in the present study, deliberate pesticide poisoning was found to be the most frequent method. Similar findings were reported earlier from Sagar island of the Sundarban region.[10] This study and the concurrent participatory observations in this region revealed some interesting findings that need attention. These are listed as follows:

### Availability of pesticides:

Agriculture is the main livelihood of people in the Sundarban region. However, Sundarban is well-known for its saline soil and varieties of pests and insects. An inverse relation was observed between the increase in population and a decrease in per-capita cultivable land in successive years. To compensate for the low yield of crops, aggressive use of fertilizers, pesticides and insecticides has been the general rule for the farmers in the region. This invites easy availability of different varieties of organophosphorous pesticides through an unregulated, open local market. Most of the farmers lack proper education about storage and use of these lethal chemicals. They keep pesticides carelessly in their home within easy access of other family members including children. There is also no control on the sale or purchase of pesticides. Numerous unregistered pesticide shops are available in the entire region. Pesticides are sold even from grocery shops in the villages. To cite an example, the small island of Sagar has 114 registered and over 210 unregistered pesticide shops.[10]

The present study found that ‘unknown poisoning’ was responsible for a good number of DSH cases. Unregistered shops sell various kinds of pesticides that are recognized only by local brand names, not by the chemical compositions. This causes a practical difficulty for the physicians at the BPHC to identify the poison and treat those poisoning cases. Thus, some restrictions and instructions for safe use and custody are imperative to reduce the burden of pesticide-related morbidity and mortality in the region. Restriction on the availability of lethal pesticides is now considered as one of the cornerstone approaches in suicide prevention.[11–13]

### Psychosocial stressors and gender specificity:

Reduction of psycho-social stressors constitutes an important strategy in suicide prevention in developing countries.[14] The number of female suicide attempters exceeded that of the male attempters. The FGDs with the Panchayat Samities revealed that different gender-specific causes are responsible for DSH attempts by females such as dowry demands, torture, mental and physical humiliation by the in-laws, derogatory behavior by alcoholic husbands or emotional or economic distress resulting from extra-marital relations of the husbands.

On the other hand, failure in examinations or love affairs and economic hardship were more common causes in males inflicting DSH. It is also stated that in most of the female attempters, there were no intentions to die; rather they used pesticides in a manipulative way to enforce threats on their family members to overcome distressing situations that they could not negotiate in a normal way. Examinations and interviews of many DSH cases in the field are consistent with this revelation. In this sense, DSH in some cases acted as a cultural mode of communication that followed the mechanism of hysterical symptom formation. The standard of living is very poor in the region and most of the families have some form of economic distress.

The dowry system is widely prevalent and dowry demand was one of the most important causes of familial conflict. An interesting comment came from an FGD with a Panchayat member of Namkhana block who admitted that self-poisoning by pesticide has become a fashion in the entire Sundarban region and this popularity of pesticides is fast replacing the traditional methods (like hanging or immolation) of self-harm in recent years. According to him, whenever people are subjected to some stressful life situations, they tend to ingest pesticide either to die or for a change (in the familial relationship dynamics) that is profitable to them. Meetings with pesticide shop owners revealed that quite often females or children went to the shops to buy pesticides as instructed by their parents. This type of practice also makes ready accessibility to lethal poisons even when it is not available in the home.

### Lack of education on safe use of pesticides:

Information regarding potential health hazards of pesticides is nonexistent in the entire area. Most of the farmers never use masks, gloves and eye-protectors while spraying pesticides in the field. Nonfunctioning and complete inattention of the local agricultural office in this regard is a serious drawback. It is felt that the easy availability of these compounds acts as a catalyst to facilitate self-harm among vulnerable subjects. Easy access to pesticide poisons was also found to be linked with DSH motives and high impulsivity.[5]

Agriculture is the most common occupation in our country and agrochemical pesticides were associated with high morbidity and mortality in the DSH cases, particularly from rural areas.[9] Therefore, an urgent intervention program is needed to reduce this preventable morbidity and mortality.

An intersectoral program involving local agricultural offices (for farmers' education on safe use and custody and health hazards of pesticides) and BPHCs (community awareness and psychosocial intervention) is the ideal goal for a community mental health program in this rural region. Restricted use and sale of pesticides,[15,16] improved awareness regarding safe custody of pesticides, using less harmful compounds in agriculture, rules for banning unlicensed shops that sell pesticides illegally may all be effective and should be encouraged. Public health advocacy to prevent poisoning by indigenous substances like yellow oleander seeds is also warranted.

At the same time, improved hospital infrastructure for proper medical care of poisoning cases[17] and timely psychosocial intervention at the community level will be beneficial. The study has some limitations: 1) Canning II, Basanti and Gosaba show very poor documentation of data mainly because of improper hospital infrastructure in those remote rural areas, 2) Not all cases of DSH attended hospitals for various reasons. Therefore, the actual number is well in excess of what is recorded in this study.
